# Preference and Willingness to Pay for the Regular COVID-19 Booster Shot in the Vietnamese Population: Theory-Driven Discrete Choice Experiment

**DOI:** 10.2196/43055

**Published:** 2023-01-31

**Authors:** Bach Xuan Tran, Anh Linh Do, Laurent Boyer, Pascal Auquier, Huong Thi Le, Minh Ngoc Le Vu, Trang Huyen Thi Dang, Khuy Minh Cao, Linh Dieu Thi Le, Lam Tung Ngoc Cu, Bang Viet Ly, Duong Anh Thi Nguyen, Manh Duc Nguyen, Carl A Latkin, Roger C M Ho, Cyrus S H Ho, Melvyn W B Zhang

**Affiliations:** 1 Institute for Preventive Medicine and Public Health Hanoi Medical University Hanoi Vietnam; 2 Research Centre on Health Services and Quality of Life Aix Marseille University Marseille France; 3 SC Johnson College of Business Cornell University Ithaca, NY United States; 4 Institute of Health Economics and Technology Hanoi Vietnam; 5 Bloomberg School of Public Health Johns Hopkins University Baltimore, MD United States; 6 Department of Psychological Medicine Yong Loo Lin School of Medicine National University of Singapore Singapore Singapore; 7 Institute for Health Innovation and Technology (iHealthtech) National University of Singapore Singapore Singapore; 8 Lee Kong Chian School of Medicine Nanyang Technological University Singapore Singapore Singapore

**Keywords:** COVID-19, epidemic, vaccine, booster, willingness to take, willingness to pay, Vietnam, policy, feasibility, acceptability, infection, vaccination, social media, intervention

## Abstract

**Background:**

The COVID-19 booster vaccination rate has declined despite the wide availability of vaccines. As COVID-19 is becoming endemic and charges for regular booster vaccination are being introduced, measuring public acceptance and the willingness to pay for regular COVID-19 boosters is ever more crucial.

**Objective:**

This study aims to (1) investigate public acceptance for regular COVID-19 boosters, (2) assess the willingness to pay for a COVID-19 booster shot, and (3) identify factors associated with vaccine hesitancy. Our results will provide crucial insights into and implications for policy response as well as the development of a feasible and effective vaccination campaign during Vietnam’s waning vaccine immunity period.

**Methods:**

A cross-sectional study was conducted among 871 Vietnamese online participants from April to August 2022. An online questionnaire based on the discrete choice experiment (DCE) design was developed, distributed using the snowball sampling method, and subsequently conjointly analyzed on the Qualtrics platform. A history of COVID-19 infection and vaccination, health status, willingness to vaccinate, willingness to pay, and other factors were examined.

**Results:**

Among the participants, 761 (87.4%) had received or were waiting for a COVID-19 booster shot. However, the willingness to pay was low at US $8.02, and most participants indicated an unwillingness to pay (n=225, 25.8%) or a willingness to pay for only half of the vaccine costs (n=222, 25.4%). Although information insufficiency and a wariness toward vaccines were factors most associated with the unwillingness to pay, long-term side effects, immunity duration, and mortality rate were the attributes the participants were most concerned with during the vaccine decision-making period. Participants who had children less than 18 years old in their homes infected with COVID-19 had a lower willingness to pay (odds ratio [OR] 0.54, 95% CI 0.39-0.74). Respondents who had children under 12 years old in their family who received at least 1 vaccine dose had a higher willingness to pay (OR 2.03, 95% CI 1.12-3.66). The burden of medical expenses (OR 0.33, 95% CI 0.25-0.45) and fear of the vaccine (OR 0.93, 95% CI 0.86-1.00) were negative factors associated with the level of willingness to pay.

**Conclusions:**

A significant inconsistency between high acceptance and a low willingness to pay underscores the role of vaccine information and public trust. In addition to raising awareness about the most concerning characteristics of the COVID-19 booster, social media and social listening should be used in collaboration with health professionals to establish a 2-way information exchange. Work incentives and suitable mandates should continue to encourage workforce participation. Most importantly, all interventions should be conducted with informational transparency to strengthen trust between the public and authorities.

## Introduction

The COVID-19 pandemic has profoundly affected numerous facets of life worldwide in the past 3 years, ranging from health, well-being, and socioeconomic status to the behavioral pattern and structural characteristics of societies [1-3]. Given its effectiveness in reducing virus severity and transmission, the COVID-19 vaccine has been used as the key pandemic control measure [4,5]. According to the World Health Organization (WHO), the COVID-19 vaccination decreased the number of deaths and severe disease from COVID-19, as well as decreasing COVID-19 transmission [6]. The Centers for Disease Control and Prevention (CDC) revealed that the protection by COVID-19 vaccination is up to 90% [7]. In Vietnam, the COVID-19 vaccine was highly accepted by the general population, including high-risk groups, such as pregnant women and the elderly [8,9]. As of July 2022, 82.2% of the Vietnamese population had received at least 2 doses of the COVID-19 vaccine [10]. However, vaccine effectiveness is dependent on the individual acceptance of the vaccine as well as the type of vaccine received, lasting on average up to a year with infection-induced antibodies and up to 6 months with vaccine-induced antibodies [11-14]. The resurgence of COVID-19 can be caused by several factors, including the decline in immunity to the COVID-19 vaccine, which emphasizes the importance of booster shots. Devenport et al [15] revealed that every 108 days or so, persons inoculated against COVID-19 would lose almost half of their protective antibodies [15]. Hence, immunizations that provided 90% protection against mild episodes of illness may only be 70% effective after 6 or 7 months [15]. Immunological studies have also shown a steady decline in antibody levels in people who have been vaccinated against COVID-19, and suggest an increased risk of an infection outbreak [16,17].

Given the effectiveness of COVID-19 booster shots, the current health focus of COVID-19 worldwide has shifted from disease control to booster regularity and long-term maintenance [18-21]. As COVID-19 progresses to its endemic phase, a pattern observed in previous major pandemics, the disease will likely not be eradicated but instead will be controlled by regular vaccinations [22,23]. Therefore, the promotion of booster vaccination is a key intervention in the new response scheme. However, unlike mandatory doses, the current COVID-19 booster shots record relatively low acceptance, even in the biggest COVID-19 vaccine–supplying countries, such as India (55.9%) [24] or the United States (61.8%) [23]. China’s contribution to the COVID-19 vaccine cannot be ignored. Although China is among the top countries with the highest acceptance of the COVID-19 vaccine in the world [25], the acceptance to take COVID-19 booster shots rate has dropped to only 76.8% [26]. In Vietnam, this rate is below 60% compared to nearly 95% coverage for mandatory shots [10]. Many countries have also experienced a recent halt in booster administration rates due to concerns about their long-term effects and importance. In the coming endemic phase, the current vaccine policy in Vietnam will undergo major revisions, the most concerning of which is the transition from free-of-charge vaccines to with-charge and regular vaccination. Therefore, assessing the willingness of the population to pay for COVID-19 boosters is crucial to inform policy makers and public health practitioners on how to proceed with and promote vaccination costs.

Several frameworks have strived to describe the correlations between different factors for the acceptance of vaccines, most commonly the integrated framework of the Health Belief Model (HBM) and the theory of reasoned action (TRA) [27,28]. Indicators in this model have been consulted, such as the perception of severity and benefits, as well as the limitations of vaccination, attitude, and subjective norms, during previous pandemics [29]. During the COVID-19 outbreak in Malaysia, Ng et al [30] added contextual factors, such as susceptibility, trust, and vaccine preference, to improve the applicability of this framework to COVID-19 vaccine acceptance [30]. Integrated models were perfected through pandemics and effectively used to inform COVID-19 vaccine campaigns. However, the acceptance of initial shots and booster shots differs immensely in that multiple doses of COVID-19 boosters are required over time and depend on individual progress instead of single or 2-dose mass public vaccinations. Thus, conventional vaccine acceptance models cannot be easily applied to understand booster shot hesitancy [31].

Moreover, the effectiveness and side effects of booster shots have been well documented. Therefore, one’s decision to receive a booster is influenced by a personal preference for known vaccine characteristics rather than a hypothetical evaluation of the vaccine’s severity, benefits, or public attitude, as suggested by previous frameworks. This change in the nature of predictors calls for a new approach to understanding vaccine acceptance: to investigate personal priorities for vaccine characteristics and assess the extent to which one is willing to take or pay in acknowledgment of risks.

Although the initial acceptance of the COVID-19 vaccine has been well studied for 3 years, little is known about the public’s willingness to pay due to the free-of-charge policy for the COVID-19 vaccine in most countries worldwide and in Vietnam [26,32,33]. However, given current economic damages, it is unlikely that COVID-19 boosters can be provided under the same policy. Thus, the measurement of the willingness to pay for a COVID-19 booster has become a priority to inform an important transition of the vaccine implementation approach. We conducted this study during Vietnam's waning vaccine immunity period to (1) investigate public acceptance for regular COVID-19 boosters, (2) assess the willingness to pay for a COVID-19 booster shot, and (3) identify factors associated with vaccine hesitancy. Our results will provide crucial insights into and implications for policy response as well as the development of a feasible, effective, and sustainable vaccination campaign.

## Methods

### Study Design

The PREVENT (Preference For Vaccine Evaluation & Trail) study was Vietnam’s nationwide assessment using a patient-centered design to inform health technology development and acceleration. Designed using the Qualtrics system, the PREVENT study included an online interactive questionnaire to target subjects across different regions of Vietnam from April to August 2022. Respondents were Vietnamese who live in Vietnam, who were aged 16 years and above (in Vietnam, people from the age of 16 years must take responsibility for their actions; moreover, they must bear penal liability like adults), and who were being referred to and agreed to complete the survey. The PREVENT study included 2 groups: (1) the general population and (2) health professionals and medical students. Snowball sampling was used for disseminating the survey, involving 20 “seeders” in all 3 regions: northern, middle, and southern. Participants took 30 minutes to complete the questionnaire and were encouraged to introduce more acquaintances and colleagues to the survey. Respondents were able to review and change their answers through a Back button in the Qualtrics system.

The PREVENT study included 5 topics of interest: (1) COVID-19 vaccine booster for adults, (2) monkeypox, (3) COVID-19 vaccine for children, (4) HIV vaccine, and (5) a hypothetical pandemic in the future. After answering general social demographic questions, each respondent was randomly assigned to 1 of these 5 topics, generating 5 separate data sets. In total, 5700 respondents were included in the PREVENT study. The substudy of the COVID-19 vaccine booster for adults included 871 complete records.

### Ethical Considerations

Participants were informed of the benefits and risks of participating in this study, and they provided informed consent. Records were monitored and tracked using IP addresses using the Qualtrics system to ensure the validity of the data set; they were then extracted, analyzed, and stored safely and confidently and used merely for research purposes. The protocol was approved by ethical review committees designated by the Vietnam Ministry of Health (decision no. 164/GCN-HDDDNCYSH-DHYHN and 13/HDDD-DHDT).

### Measurement and Instrument

We applied a standard procedure for generating the research instrument. Initially, a systematic review was conducted to identify important facets that emerged from previous studies. Next, we constructed the questionnaire, covering the breadth of measurements of interest. A group of experts in public health, infectious diseases, health services, econometrics, linguistics, representatives of target groups, and research assistants joined in the deliberation of translating, rephrasing, piloting, and shortening the questionnaire. Finally, the tool included 5 major sessions: (1) sociodemographics, (2) a history of COVID-19 infection and vaccination, (3) the willingness to take a COVID-19 vaccine booster for adults, and (4) the willingness to pay for it.

### Outcome Variables

#### Willingness to Vaccinate

A short questionnaire was designed to determine the participants' willingness to take the COVID-19 vaccine booster for adults using the following 3 items:

- Will not vaccinate
- Intend to inject COVID-19 booster shots
- Have completed the COVID-19 booster shots

#### Willingness to Pay for the Vaccine

A questionnaire was designed to determine the participants' willingness to pay for vaccines using the following 5 options:

To what extent are you willing to pay for the vaccine?

- Unwilling to pay
- 20% of the cost
- 50% of the cost
- 80% of the cost
- Full cost

The actual cost of the vaccines was not known to participants. This question was used to examine the participants’ willingness to pay when financial support/a discount was provided.

#### Socioeconomic Status

Participants responded to questions about their sociodemographics, including age, gender (male/female), marital status (single, others), job, monthly household income per capita, and area.

#### History of COVID-19 Infection and Vaccination

Participants responded to questions about their personal and family history of COVID-19, time since the last COVID-19 infection, health status, and personal and family history of COVID-19 vaccination.

#### Factors Affecting Vaccination to Prevent Disease

We used 10 items corresponding to 2 factors to measure the factors affecting vaccination to prevent disease. A 3-point Likert scale was used to evaluate the answers.

Factor 1: concerns about the vaccine and responsibility to the community

- Item 1. Concerned that the vaccine is newly developed.
- Item 2. Concerned about immediate side effects of the vaccine.
- Item 3. Concerned about long-term side effects of the vaccine.
- Item 4. Concerned about new components of the vaccine.
- Item 5. Concerned about the immunity duration of the vaccine.
- Item 9. Vaccinate so the community can maintain normal living and working conditions.
- Item 10. Vaccinate to fulfill personal responsibility of disease prevention.

Factor 1 included 7 questions. The total score was calculated by adding the scores of the 7 items and then converted to a 10-point scale.

Factor 2: fear of the vaccine

- Item 6. Fear of vaccines and injections in general.
- Item 7. Fear of insufficient information to make decisions.
- Item 8. Wait for others to vaccinate first.

Factor 2 included 3 questions. The total score was calculated by adding the scores of the 3 items and then converted to a 10-point scale.

Hence, the score of factors 1 and 2 ranged from 1 to 10. Participants with a higher score indicated a heightened concern about the vaccine and responsibility to the community and a heightened fear of the vaccine. The Cronbach α of factors 1 and 2 was good at .89 and .84, respectively.

#### Interpersonal Factors

We included 8 items to assess the interpersonal factors related to vaccination. A 3-point Likert scale was used to evaluate the answers. There were 2 domains included:

Factor 1: risks of infection and fear of the impact of the disease on health and economy

- Item 1. How is the risk of reinfection for you and your family members?
- Item 2. How is the risk of reinfection for children in your family?
- Item 3. How afraid are you of the spread of this disease?
- Item 4. Ho afraid are you of the impact on the health of this disease?
- Item 5. How afraid are you of the impact on the economy of this disease?

Factor 2: service satisfaction

- Item 6. How satisfied are you with vaccination services?
- Item 7. How satisfied are you with COVID-19 consultation services?
- Item 8. How satisfied are you with health care services for pandemics?

After summing the total score of each item, the total score of factors 1 and 2 was converted to a 10-point scale. Respondents with a higher score indicated a higher risk of infection and fear of the impact of the disease on health and economy or service satisfaction. The Cronbach α of factors 1 and 2 was good at .87 and .91, respectively.

### Discrete Choice Experiment

We conducted a literature review of factors affecting the willingness to take and to pay for vaccination services and the designs of previous discrete choice experiments (DCEs) on adult vaccination. The results informed our selection of 6 major attributes of such services that influence an individual's preference, including effectiveness (<60%, 60%-90%, and >90%), immunity duration (3-6 months to a lifetime), side effects (minor [can maintain daily functioning] to major [severe fatigue and immobility]), mortality rate (0.001%-0.01%), limitations if not vaccinated (yes or no), cost of the vaccine (VND 100,000 [US $4.27] to VND 2,000,000 [US $85.33]) [34-39]. These attributes were then assigned 2-5 levels for choosing, contributing to a total of 31 × 41 × 23 × 51 = 480 possible alternatives (Table 1). Each participant was asked to respond to 7 different scenarios based on generated combinations by selecting which scenarios they preferred.

The sample size for the DCE was determined using Sawtooth software to determine the number of responses: sample size = (multiplier*c)/(t*a) = 357, where multiplier=1000, “c” is the largest number of levels across all features (n=5), “t” is the number of tasks or questions (n=7), and “a” is the number of alternatives or choices per question (n=2). This sample size was reached for both target groups of the survey, with 461 (52.9%) in the general population and 410 (47.1%) in health professionals and medical students.

**Table 1:** Vaccine attributes in the DCE^a^.

Attributes	Response options
Effectiveness (%)	<60 60-90 >90
Immunity duration	3-6 months 6-12 months 1-3 years Lifetime
Side effects	Minor: can maintain daily functioning Major: severe fatigue and immobility
Mortality rate	0.001% 0.01%
Limitations if not vaccinated	No Yes: traveling banned and social gatherings restricted
Cost (VND/US $)^b^	100,000/4.27 200,000/8.53 500,000/21.33 1,000,000/42.67 2,000,000/85.33

^a^DCE: discrete choice experiment.

^b^VND 23,437.98=US $1.00.

### Statistical Analysis

Statistical analysis was performed using Qualtrics and STATA software (version 15). Descriptive data were generated for all variables. With missing data, we used the listwise deletion method to clean data before analyzing them; 1071 records were collected, of which 871 (81.3%) records were complete, so 200 (18.7%) records were excluded from the analysis. Continuous variables were presented as the mean (SD), while categorical variables were presented as frequencies with percentages.

### Factorial Structure

Exploratory factor analysis (EFA) using principal component analysis (PCA) was performed to evaluate the optimal structural model of the instrument according to the observed data. The number of factors was determined based on the Scree plot, and parallel analysis, along with eigenvalues and the proportion of variance explained (Multimedia Appendices 1 and 2). Items with a loading value of ≥0.5 were included in the relevant component.

Potential covariates for full models of the decision to take and the willingness to pay for COVID-19 boosters for adults included socioeconomic data, COVID-19 characteristics, related information regarding the COVID-19 vaccine, factors affecting the intention to vaccinate for disease prevention, and interpersonal factors (Multimedia Appendices 1 and 2). We used multivariate ordered logistic regression to identify factors related to the willingness to pay for COVID-19 booster and multinomial logistic regression to identify factors associated with the willingness to take a COVID-19 booster for adults. *P*<.05 was considered statistically significant.

In DCE data analysis, individual-based utility models were yielded using hierarchical Bayes estimation that uses Bayesian methods to probabilistically derive the relative value of each tested variable. The models estimated the optimal package of the preferred attributes of COVID-19 vaccination and the contribution of each attribute.

## Results

### Participant Characteristics

Table 2 demonstrates the demographic characteristics of the respondents. More than two-thirds of the participants (n=607, 69.7%) were females, and 661 (75.9%) were aged from 16 to 24 years. Most respondents finished college, university, postgraduate education (n=702, 80.6%) and were not married (n=707, 81.2%). Health care professionals and medical students (n=410, 47.1%) constituted the largest occupational group. The mean average monthly income per household was VND 3-10 million (US $128.00-$426.66; n=337, 38.7%).

**Table 2:** Demographic characteristics of respondents (N=871).

Characteristics			Participants, n (%)
**Gender**			
	Male	264 (30.3)	
	Female	607 (69.7)	
**Age group (years)**			
	16-19	305 (35.0)	
	20-24	356 (40.9)	
	>25	210 (24.1)	
**Education level**			
	Not graduated from high school	95 (10.9)	
	Graduated from high school	74 (8.5)	
	College/university/postgraduate	702 (80.6)	
**Marital status**			
	Single/divorced/widowed	707 (81.2)	
	Married	164 (18.8)	
**Job**			
	Health care worker/medical student	410 (47.1)	
	Other students	190 (21.8)	
	Stable jobs	64 (7.3)	
	Other jobs	207 (23.8)	
**Monthly household income per capita (VND/US $)**			
	<1 million/<42.67	249 (28.6)	
	1-2.9 million/42.67-123.73	175 (20.1)	
	3-10 million/128.00-426.66	337 (38.7)	
	>10 million/>426.66	110 (12.6)	
**Children**			
	No children	710 (81.5)	
	Pregnant/have children	161 (18.5)	
**Area**			
	Hanoi	391 (45.0)	
	Other northern provinces/cities	102 (11.7)	
	Southern provinces/cities	101 (11.6)	
	Central provinces and Central Highlands	192 (22.1)	
	Other provinces	85 (9.6)	

Table 3 presents the history of previous infections, vaccinations, and health characteristics of the respondents. Of 871 participants, 492 (56.3%) were infected with COVID-19 and 249 (28.5%) had children in the family infected with COVID-19. The most prevalent infection period was the recent 3-6 months. The largest proportion of participants (n=400, 46.1%) reported a moderate health status, followed by 359 (41.4%) who self-reported that they were completely healthy. Regarding the COVID-19 vaccination for self and family, two-thirds of the participants had taken the third, or booster, dose (n=582, 66.7%), while only 46 (5.3%) participants had children below 12 years old in their family who had been vaccinated. The most popular information sources were health care officials (n=592, 67.8%) and the media (n=457, 62.7%).

**Table 3:** Personal and family history of COVID-19 infection and vaccination of study subjects (N=871).

Characteristics		Participants, n (%)
**Personal and family history of COVID-19**		
	Nobody in my family has been infected COVID-19.	128 (14.7)
	I was infected with COVID-19.	492 (56.3)
	Adults in my family were infected with COVID-19.	481 (55.1)
	Children <18 years old in my family were infected with COVID-19.	249 (28.5)
**Time since the last COVID-19 infection**		
	Not yet infected	381 (43.7)
	1-3 months	154 (17.7)
	3-6 months	283 (32.4)
	>6 months	54 (6.2)
**Health status**		
	Completely healthy (100%)	359 (41.4)
	Relatively healthy (80%-100%)	400 (46.1)
	Slightly compromised health (<80%)	109 (12.5)
**History of COVID-19 vaccination of self and family**		
	I have received 2 doses.	213 (24.4)
	I have received 3 doses or a booster or both.	582 (66.7)
	Children under 12 years old in my family have received at least 1 vaccine dose.	46 (5.3)
	Children 12-17 years old in my family have received at least 1 vaccine dose.	105 (12.0)
	All adults in my family have received at least 2 doses.	436 (49.9)
**Vaccine information sources**		
	Health care officials	592 (67.8)
	Relatives, friends, neighbors	371 (42.5)
	Media (health consultation switchboard, radio, newspaper, television)	547 (62.7)
	Others	77 (8.8)

### Willingness to Take and Willingness to Pay for a COVID-19 Booster

Table 4 and Multimedia Appendix 3 demonstrates the respondents' willingness to receive and their reasons for vaccine refusal. Nearly two-thirds of the participants received a booster shot (n=524, 60%), and only 61 (7%) participants did not want a booster vaccine. The most common reasons for vaccine hesitancy included insufficient time since the last shot (n=38, 34.6%) and the wait-and-see approach (n=31, 28.2%). In addition, 225 (25.8%) respondents were unwilling to pay for a COVID-19 booster, and 222 (25.4%) were willing to pay half of the vaccine cost (ie, the other half of the cost had to be covered by someone else). A considerable proportion (n=571, 65.6%) viewed the booster shot as an economic burden.

**Table 4:** Willingness to take and willingness to pay for a COVID-19 booster.

Characteristics			Participants, n (%)
**Willingness to take a COVID-19 booster**			
	Will not vaccinate	61/871 (7.0)	
	Intend to inject COVID-19 booster shots	288/871 (33.0)	
	Have completed COVID-19 booster	524/871 (60.0)	
**Reasons for refusal to vaccinate**			
	Not enough time between COVID-19 shoots	38/109 (34.6)	
	Feeling unwell	9/109 (8.2)	
	Have underlying medical problems or other medical treatment	6/109 (5.5)	
	Allergic history	3/109 (3.6)	
	Am waiting for my turn to be vaccinated	14/109 (12.7)	
	Wait and see	31/109 (28.2)	
	No means of transport and money to travel to the place of injection	0	
	Suffered from severe side effects of the previous injection	17/109 (15.5)	
	Feel that booster is unnecessary as COVID-19 has become irrelevant	29/109 (26.4)	
	Have just been infected	28/109 (25.5)	
**Willingness to pay for a COVID-19 booster**			
	Unwilling to pay	225/871 (25.8)	
	20% of the cost	133/871 (15.2)	
	50% of the cost	222/871 (25.4)	
	80% of the cost	93/871 (10.7)	
	Full cost	200/871 (22.9)	
**Financial burden of COVID-19 booster**			
	No	300/871 (34.4)	
	Yes	571/871 (65.6)	

### Multivariable Analyses to Identify Factors Associated With the Decision to Take and the Willingness to Pay for a COVID-19 Vaccine

Table 5 reveals the factors associated with the decision to take the COVID-19 vaccine. People who had higher education (odds ratio [OR] 17.22, 95% CI 2.86-103.63), who had graduated from high school (OR 12.35, 95% CI 3.46-44.00), who had received 3 doses or a booster or both (OR 13.31, 95% CI 4.31-41.09), and in whose family all adults had received at least 2 doses (OR 2.88, 95% CI 1.31-6.31) were likely to have completed the COVID-19 booster. Relatively healthy participants were likely to intend to get the COVID-19 vaccine (OR 2.98, 95% CI 1.37-6.47).

Multivariate ordered logistic regression results to identify factors associated with the willingness to pay for the COVID-19 vaccine are presented in Table 6. Participants aged 20-24 years, who had a monthly household income per capita of >VND 10 million, in whose family adults were infected with COVID-19, in whose family children under 12 years old had received at least 1 vaccine dose, who used media to access information about the vaccine, and who had higher concerns about vaccine characteristics and responsibility to the community were likely to have a higher level of willingness to pay for the COVID-19 vaccine. Children <18 years old in the family infected with COVID-19 and the burden of medical expenses were harmful factors that reduced the level of willingness to pay for the COVID-19 vaccine.

**Figure 1:**
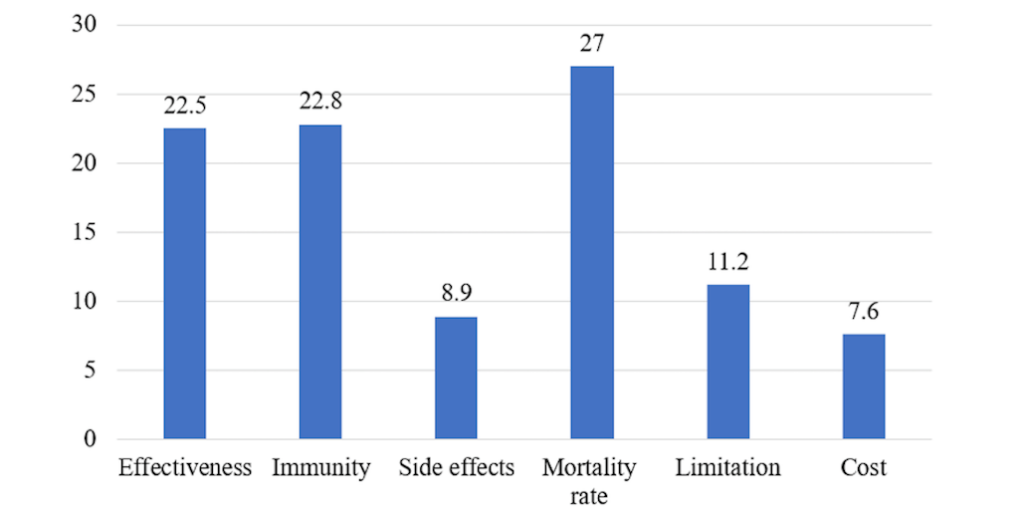
Feature importance of vaccine attributes during the decision-making process.

Figure 1 presents the feature importance of each vaccine attribute generated by the conjoint analysis. The mortality rate had the most influence on the decision-making process, at 27 points, followed by immunity at 22.8 points and vaccine effectiveness at 22.4 points. The cost was the least weighted attribute, at 7.6 points.

Table 7 shows the willingness to pay based on current COVID-19 vaccine attributes: more than 90% effectiveness, 6-12 months’ immunity, minor side effects, insignificant mortality rate, and major limitations if not vaccinated. For this package, the generated willingness to pay was VND 188,000 (US $ 8.02).

**Table 5:** Factors associated with the decision to take the COVID-19 vaccine.

Factors		Willingness to get the vaccine	
		“Will not vaccinate” vs “have completed the COVID-19 booster,” OR (95% CI)	“Intend to inject” vs “have completed the COVID-19 booster,” OR (95% CI)
**Education level (reference: not graduated from high school)**			
	Graduated from high school	0.06^a^ (0.01-0.35)	0.20^b^ (0.06-0.68)
	College/university/postgraduate	0.08^a^ (0.02-0.29)	0.21^a^ (0.07-0.60)
**City/province (vs Hanoi: reference)**			
	Northern provinces	0.69 (0.20-2.41)	1.04 (0.57-1.91)
	Southern provinces	1.65 (0.60-4.55)	0.33^a^ (0.16-0.68)
	Central provinces and Central Highlands	0.72 (0.27-1.94)	0.70 (0.41-1.21)
	Other provinces	0.19^c^ (0.03-1.06)	0.59 (0.29-1.23)
**Personal and family history of COVID-19**			
	Children <18 years old in my family were infected with COVID-19.	1.71 (0.76-3.81)	1.77 ^b^ (1.12-2.79)
**Health status (vs completely healthy 100%: reference)**			
	Relatively healthy (80%-<100%)	0.50^c^ (0.22-1.12)	1.48^c^ (0.97-2.27)
	Slightly compromised health	2.44^c^ (0.92-6.45)	2.18^b^ (1.17-4.05)
**History of COVID-19 vaccination for yourself and your family (yes vs no: reference)**			
	I have received 2 doses.	7.03^a^ (2.46-20.06)	5.76^a^ (3.00-11.04)
	I have received 3 doses or a booster or both.	0.08^a^ (0.02-0.23)	0.14^a^ (0.08-0.24)
	All adults in my family have received at least 2 doses.	0.35^a^ (0.16-0.76)	0.38^a^ (0.24-0.60)

^a^*P*<.10.

^b^*P*<.05.

^c^*P*<.01.

**Table 6:** Factors associated with the willingness to pay for the COVID-19 vaccine.

Factors		Level of willingness to pay (1=unwilling to pay to 5=willing to pay the full cost), OR (95% CI)
**Age (years; reference: 16-19 years)**		
	20-24	1.40^a^ (1.03-1.91)
	>25	1.20 (0.77-1.87)
**Job (reference: health care worker/medical student)**		
	Other students	1.07 (0.77-1.50)
	Stable jobs	1.41 (0.77-2.58)
	Other jobs	1.70^b^ (1.14-2.53)
**Monthly household income per capita (VND/US $; reference: <VND 1 million/<US $42.67)**		
	1-2.9 million/42.67-123.73	0.88 (0.61-1.29)
	3-10 million/128.00-426.66	1.22 (0.87-1.71)
	>10 million/>426.66	2.36^b^ (1.47-3.79)
**Personal and family history of COVID-19 (vs nobody has been infected with COVID-19: reference)**		
	Adults in my family were infected with COVID-19.	1.33^a^ (1.01-1.77)
	Children <18 years old at my family were infected with COVID-19.	0.54^b^ (0.40-0.74)
**History of COVID-19 vaccination for yourself and your family (yes vs no: reference)**		
	Children under 12 years old in my family have received at least 1 vaccine dose.	1.94^a^ (1.09-3.48)
**Medical expense burden (** **yes vs no: reference** **)**		
	The burden of medical expenses	0.33^b^ (0.25-0.45)
**Resources to access information on vaccine (yes vs no: reference)**		
	Media (health consultation switchboard, radio, newspaper, television)	1.36^a^ (1.03-1.80)
**Factors affecting vaccination to prevent disease**		
	Concerns about vaccine characteristics and responsibility to the community (unit: score)	1.10^a^ (1.01-1.20)
	Fear of the vaccine (unit: score)	0.93^c^ (0.86-1.00)
**Interpersonal factors**		
	Service satisfaction (unit: score)	1.05 (0.98-1.13)

^a^*P*<.05.

^b^*P*<.10

^c^*P*<.01.

**Table 7:** Willingness to pay for a hypothetical COVID-19 vaccine booster for adults generated by the DCE^a^.

Vaccine attribute	Value
Vaccine effectiveness	More than 90%
Immunity	6-12 months
Side effects	Minor, does not affect daily functioning
Mortality rate (/100,000 people)	1 death
Limitation if not vaccinated	Yes: traveling banned and social activity restricted
Cost (VND/US $)	188,000/8.02

^a^DCE: discrete choice experiment.

## Discussion

### Principal Findings

This study results indicated the participants' high willingness to take COVID-19 vaccine booster shots. Their willingness to pay, however, was scattered between price levels, the most common of which were willingness to pay the full price, willingness to pay half the price, and total unwillingness to pay at all. The reasons for an unwillingness to pay and booster hesitancy were concerns of long-term effects, immunity duration, information insufficiency, lack of confidence in the vaccine, unawareness of the risk of reinfection, and dissatisfaction with COVID-19 care services. Based on the identified issues of lack of information and weakening public trust in the vaccine, we proposed personal, interpersonal, and managerial solutions to ensure affordable vaccinations and consistent uptake. The burden of medical expenses and the fear of vaccines were negative factors associated with the willingness to pay; in contrast, participants who had children under 12 years old in their family who had received at least 1 vaccine dose used the media to access information about vaccines, while concerns about vaccine characteristics and responsibility to the community had the opposite impact.

In our study, 87% of the participants had taken a booster dose or were waiting to get vaccinated, which is generally higher than that in low-, middle-, and high-income countries (73.4% in low-income countries, 67.9% in middle-income countries, and 83.0% in high-income countries) [40].

When examined at the national level, the willingness to pay generated in our study accounted for a small fraction of the per capita monthly income of our sample (VND 3,000,000-10,000,000, or US $ 128.00-426.66). Since the effects of the COVID-19 vaccine typically start to diminish after 6 months, the actual monthly cost of a booster is even lower when spread out over months, making it a low financial burden. Indeed, a critically low willingness to pay despite a high acceptance rate might be a proxy for people’s underestimation of vaccine importance, suggesting gaps in communication between the general public and authorities. This is consistent with the fact that the most common reason for vaccine hesitancy was a lack of knowledge about the vaccine.

Public information about COVID-19 boosters does not cover the main factors highlighted by this study that influence the vaccine decision and willingness to pay, such as potential side effects, mortality rate, induced immunity, and effectiveness. Furthermore, as health care professionals and the media were deemed to provide the most trusted information, public education approaches should be more comprehensive and interactive, allowing for televised question-and-answer (Q&A) sessions with experts, hotline services staffed by trained practitioners, and open informational portals made available on social media. Social listening is another recommended approach to be used by governments and health organizations to understand people’s perceptions and concerns, to refute false information and handle controversies, and to identify other social leaders or influencers who may have a positive impact on the general public.

Our findings emphasize the importance of building public trust when disseminating information about vaccinations. Contrary to traditional vaccines with a long history of development and trials, the COVID-19 vaccine was made available on an emergency warranty after preclinical studies and only 12-18 months of multiphase trials [41]. Despite ongoing efforts to map the long-term effects of the COVID-19 vaccine, the lack of longitudinal evidence leaves them unconfirmed to both clinicians and the general public. Although rarely, the COVID -19 vaccine can also have adverse side effects, such as myocarditis, Guillain-Barre syndrome, or thrombotic syndrome [42]. This information, if not communicated clearly and transparently, may cause public distrust, which in turn undermines the national sustainable vaccine uptake [43]. Making COVID-19 vaccinations a regular/seasonal requirement may also prove challenging in the absence of public transparency. During the COVID-19 outbreak, it was evident that public trust differed between the types of response, specifically between countries whose governments showcased true surveillance results and those whose governments intentionally provided incomplete information in an attempt to comfort the public [44,45]. The widespread availability and ease of use of social media platforms hastened the spread of false information online. This issue is further exacerbated if there exists low vaccine literacy in the country [46]. Public distrust, if it already exists, can be tackled through social listening, educating, and engaging with advocates, as in the case of the influenza outbreak [47].

Other significant factors promoting vaccine acceptance were fear of disrupting normal working and living conditions, reinfection, and re-emergence of the outbreak. In the work sphere, various interventions can be implemented to improve vaccine uptake, including paid time off for vaccinations or recovery time. A study among US workers observed that such incentives could improve vaccination rates, and they have been frequently implemented since the beginning of vaccination programs [48]. Financial plans that allow the cost of the COVID-19 vaccine to be deducted directly from one’s salary and the payments to be spread over months should be encouraged to relieve the financial burden. The inclusion of the booster vaccine in the standard workplace health requirements or routine health check-ups must be endorsed by relevant institutions. Since COVID-19 has been identified as a massive public burden affecting the entire economy, the costs associated with its prevention could be shared by both public and private sectors.

Interventions drawing on the fear of a COVID-19 pandemic re-emergence are numerous. Nudge interventions, such as mobile phone personal reminders or fixated appointments, have been found to be effective in increasing vaccine uptake in Denmark [49]. In Vietnam, a new “Vaccine Reminder” extension can be added to the national COVID-19 portal, which was made available and effectively used for case surveillance during the pandemic peak. Restrictions regarding social gatherings and traveling should continue to be imposed to make boosters informally mandatory [50].

### Strengths and Limitations

The strengths of our study included a large sample size and diverse demographics, which enabled high generalizability. Our DCE design and conjoint analysis allowed us to imitate real-life conditions of the willingness to pay and generate a more reliable result.

Numerous limitations also exist, including the inability to infer causal relationships due to a cross-sectional design, the influence of the current vaccine policy on respondents’ perception, and personal bias due to self-reporting. Nonetheless, this study is 1 of the first and most significant in our country to provide early insights into the public acceptance and willingness to pay for COVID-19 boosters, thus making substantial contributions to Vietnam's efforts to integrate COVID-19 boosters into its regular health care requirements.

### Conclusion

Our study showed a high willingness to take yet a low willingness to pay for a COVID-19 vaccine booster among Vietnamese participants. More importantly, the low willingness to pay was traced to insufficient information and underestimating vaccine importance. To resolve the lack of knowledge, we proposed a vaccine promotion approach with emphasis on side effects, mortality rate, immunity duration, and effectiveness, as well as social listening to counter misinformation. As the media and health experts are the most trusted information providers, their role in monitoring, directing, and resolving issues of public perception can be optimized through interactive interventions, such as Q&A sessions between influential health figures on television or an online platform, a COVID-19 vaccine–specialized hotline by health officials, and use of social listening insights. Work incentives, such as assistance for COVID-19 vaccine sick leave and technical convenience for vaccine reminders, can also be provided to encourage vaccine uptake. Above all, all vaccine promotion campaigns should be based on an open display of benefits as well as unwanted limitations of vaccines to achieve a nationally accepted regular COVID-19 vaccination in the long term.

## Appendix

Multimedia Appendix 1 Exploratory factor analysis about factors affecting vaccination to prevent disease.

Multimedia Appendix 2 Exploratory factor analysis of interpersonal factors.

Multimedia Appendix 3 Willingness to take and willingness to pay for a COVID-19 booster regarding 2 groups.

## Abbreviations

DCE: discrete choice experiment
OR: odds ratio
