# Supplementary material for: Preference and Willingness to Pay for the Regular COVID-19 Booster Shot in the Vietnamese Population: Theory-Driven Discrete Choice Experiment
Source: JMIR Public Health Surveill. 2023 Jan 31;9:e43055. doi: 10.2196/43055 (PMC9891355; doi:10.2196/43055)
Supplement: Multimedia Appendix 1 [file publichealth_v9i1e43055_app1.docx]

**Multimedia Appendix 1: Exploratory factor analysis about factors affecting vaccination to prevent disease**

| **Items** | **Concerns about the factor of the vaccine and responsibility to the community** | **Fear of vaccine** |
| --- | --- | --- |
| **Factors affecting vaccination to prevent disease** | | |
| 1) Concerned that the vaccine is newly developed | 0.5366 |  |
| 2) Concerned about immediate side effects of the vaccine | 0.6547 |  |
| 3) Concerned about long-term side effects of the vaccine | 0.7289 |  |
| 4) Concerned about new components of the vaccine | 0.6924 |  |
| 5) Concerned about the immunity duration of the vaccine | 0.7073 |  |
| 6) Fear of vaccines and injection in general |  | 0.743 |
| 7) Fear of insufficient information to make decision |  | 0.7696 |
| 8) Wait for others to vaccinate first |  | 0.7228 |
| 9) Vaccinate so that the community can maintain normal living and working conditions | 0.7306 |  |
| 10) Vaccinate to fulfill personal responsibility of disease prevention | 0.7332 |  |
| **Reliability** |  |  |
| Cronbach α | 0.89 | 0.84 |
| **Score (range 1-10)** |  |  |
| Mean | 6.58 | 5.28 |
| SD | 2.11 | 2.19 |

**Appendix 1** presented the exploratory factor analysis of factors affecting vaccination to prevent disease. There were 2 factors: Factor 1 “Concerns about the factor of the vaccine and responsibility to the community” had 7 items, Mean = 6.58 (SD = 2.11), and Cronbach α= 0.89; Factor 2 “Fear of vaccine” had 3 items, Mean = 5.28 (SD = 2.19) and Cronbach α= 0.84. In terms of Interpersonal factors, there were 2 factors: Factor 1 “Risks of infected diseases and fear of the impact of the disease on health and economy” had 5 items, Mean = 7.05 (SD = 2.09) and Cronbach α = 0.87; Factor 2 “Service satisfaction” had 3 items, Mean = 6.29 (SD = 2.05) and Cronbach α = 0.91 (**Appendix 2**).
