# Supplementary material for: Preference and Willingness to Pay for the Regular COVID-19 Booster Shot in the Vietnamese Population: Theory-Driven Discrete Choice Experiment
Source: JMIR Public Health Surveill. 2023 Jan 31;9:e43055. doi: 10.2196/43055 (PMC9891355; doi:10.2196/43055)
Supplement: Multimedia Appendix 2 [file publichealth_v9i1e43055_app2.docx]

**Multimedia Appendix 2: Exploratory factor analysis of interpersonal factors**

| **Items** | **Risks of infected diseases and fear of the impact of the disease on health and economy** | **Service satisfaction** |
| --- | --- | --- |
| 1) How is the risk of reinfection for you and your family members? | 0.7023 |  |
| 2) How is the risk of reinfection for children in your family? | 0.7465 |  |
| 3) How afraid are you of the spread of this disease? | 0.7462 |  |
| 4) How afraid are you of the impact on health of this disease? | 0.7360 |  |
| 5) How afraid are you of the impact on the economy of this disease? | 0.6118 |  |
| 6) How satisfied are you with vaccination services? |  | 0.7742 |
| 7) How satisfied are you with COVID-19 consultation services? |  | 0.8247 |
| 8) How satisfied are you with health care services for pandemics? |  | 0.8274 |
| **Reliability** |  |  |
| Cronbach α | 0.87 | 0.91 |
| **Score (range 1-10)** |  |  |
| Mean | 7.05 | 6.29 |
| SD | 2.09 | 2.05 |
