# Supplementary material for: Preference and Willingness to Pay for the Regular COVID-19 Booster Shot in the Vietnamese Population: Theory-Driven Discrete Choice Experiment
Source: JMIR Public Health Surveill. 2023 Jan 31;9:e43055. doi: 10.2196/43055 (PMC9891355; doi:10.2196/43055)
Supplement: Multimedia Appendix 3 [file publichealth_v9i1e43055_app3.docx]

**Multimedia Appendix 3: Willingness to take and willingness to pay for COVID-19 booster regarding** 2 **groups**

| ***Characteristics*** | **Health professionals and medical students** | | **General population** | | **Total** | | **p-value** |
| --- | --- | --- | --- | --- | --- | --- | --- |
|  | ***n*** | ***%*** | ***n*** | ***%*** | ***n*** | ***%*** |  |
| *Willingness to take COVID-19 booster* |  |  |  |  |  |  |  |
| Will not vaccinate | 15 | 3.7 | 46 | 10.0 | 61 | 7.1 | 0.002 |
| Wait and see | 19 | 4.7 | 29 | 6.3 | 48 | 5.6 |  |
| Will vaccinate when it is my turn | 117 | 28.8 | 120 | 26.2 | 237 | 27.4 |  |
| Have completed COVID-19 booster | 256 | 62.9 | 263 | 57.4 | 519 | 60.0 |  |
| *Willingness to pay for COVID-19 booster* |  |  |  |  |  |  |  |
| Unwilling to pay | 116 | 28.5 | 107 | 23.4 | 223 | 25.8 | 0.003 |
| 20% of cost | 68 | 16.7 | 64 | 14.0 | 132 | 15.3 |  |
| 50% of cost | 108 | 26.5 | 113 | 24.7 | 221 | 25.6 |  |
| 80% of cost | 46 | 11.3 | 45 | 9.8 | 91 | 10.5 |  |
| Full cost | 69 | 17.0 | 129 | 28.2 | 198 | 22.9 |  |
| *Financial burden of COVID-19 booster* |  |  |  |  |  |  |  |
| No | 124 | 31.9 | 162 | 36.7 | 286 | 34.4 | 0.148 |
| Yes | 265 | 68.1 | 280 | 63.4 | 545 | 65.6 |  |

**Appendix 3** presented that participants who have completed the COVID-19 booster had the highest percentage in both groups. Health professionals and medical students had the highest proportion of unwillingness to pay for COVID-19 boosters, meanwhile, the general population had the highest proportion of the full cost to pay for COVID-19 boosters.
